# Some Points about Head Injuries

**Published:** 1909-02-06

**Authors:** 


### Surgery.

### SOME POINTS ABOUT HEAD INJURIES.

### II.—THE TREATMENT OF CONCUSSION.

The first thing to do with a concussed patient is to put him to bed without further delay. If possible the room should be a quiet one, and it should not be brilliantly lighted: it need not be absolutely darkened; this is merely depressing. All that is necessary is to draw down the blinds, so that direct sunlight is avoided.

Hot bottles should be applied to him, especially to his feet and limbs. One word of warning is necessary. It is one of the easiest things in the world to burn an unconscious patient with hot-water bottles. To do so, however, reflects discredit both on the medical man and the nurse. The temperature of the water should therefore be carefully regulated, and the bottles themselves should be protected with flannel. A burn of this nature often produces an intractable wound. The hot-water bottle assists the patient in overcoming the shock from which he is likely to be suffering.

Stimulants, on the other hand, produce a hyperæmia which is too violent and comes on too rapidly, so that harm is done rather than good. This remark applies to strychnine and brandy, and explains the danger of administering stimulants to an injured person, a danger which was alluded to in the preceding article of this series. They should, therefore, be reserved for emergencies, such as the onset of syncope, as indicated by the pulse becoming rapid and feeble, and the breathing very shallow.

As soon as the patient is conscious, he should be given a purge. Calomel is, in our opinion, the best aperient to use in this condition. If unconsciousness is very prolonged, say for two or three days (and in a bad case this may easily occur), it is necessary to administer a purge to the patient while he is yet conconscious. In this case croton oil is a useful drug. But it is one that must be used with caution, and not lightly; for it must be remembered that it acts by setting up a more or less severe enteritis, and is, therefore, strongly contra-indicated in all cases in which any part of the alimentary canal is damaged or diseased. In the present case, however, it has undeniable advantages, because it can easily be given to an unconscious patient if two minims in a little butter are placed on the back of his tongue.

The diet should consist of milk only as a routine. If unconsciousness is unduly prolonged this may be given through a nasal tube, thirty ounces a day being sufficient for an adult.

In an ordinary case unconsciousness lasts for a few hours only; after two or three days the temperature rises to normal, the pulse rate becomes regular, and a day or two after this the patient may be regarded as convalescent. In some cases. however, the temperature remains persistently subnormal. This should be regarded as evidence that the patient is not getting enough nourishment, and the diet should be increased accordingly.

Under no circumstances should a patient who has been definitely concussed be allowed to get up before a fortnight has elapsed, and only then if he has completely recoverd from the effects of his injury. A useful method of deciding this point is to make him bend down and touch his toes several times in quick succession without bending his knees. If he can do this without feeling giddy after it he may be allowed to resume his ordinary mode of life. It is important to observe this precaution strictly, since if concussion is properly treated there should be no after effects; but if rest has not been enjoined for a sufficient length of time, persistent headache, giddiness, and loss of mental power, particularly loss of memory, are likely to ensue.

The symptoms so far described are those of an ordinary case of concussion. But in another class of case, as soon as the unconsciousness ceases, the patient passes into a state of irritability. He resents examination, and lies in bed, generally on his side, curled into a flexed position, and may even have twitchings, spasms, or convulsions. The pupils are contracted, and he refuses to take food, and often passes his excreta in the bed.

This stage varies in duration, but generally passes off in a few days, and is succeeded, as Erichsen pointed out, by a condition of fatuity.

Such symptoms point to a localised laceration of the brain substance. Autopsies on patients who have died of this disclose the fact that, although the laceration may be at a spot corresponding to the site of the injury, it is more often at a point exactly opposite, the brain having been damaged, as it is said, by *contrecoup*. The syndrome described is most often found in association with laceration of some part of the frontal lobe, the frequency of this being compatible with the fact that a large proportion of head-injuries are caused by falls upon the back of the head. A patient falling forward rarely strikes his head: he is able to protect himself by putting out his hands; but if he falls backwards, he naturally cannot do so.

The immediate result of the injury is to cause a circumscribed hæmorrhage; and in cases that recover this is gradually absorbed, and that is an end of the matter. But in others the ecchymosis is succeeded by œdema, and this spreads by pressure on the neighbouring veins, and the final result may be a localised fatty degeneration or even suppuration from a hæmic infection.

The same general principles which dictate the treatment of concussion are applicable to a case of laceration of the brain. But in this case it is well to give the patient some form of sedative, and potassium bromide in large doses is the best drug for this purpose. It is essential that he should have sufficient nourishment, but some difficulty is likely to be experienced on account of his unwillingness to take food. Otherwise the treatment is the same as that of concussion. But since laceration indicates a severer form of injury, it is well to keep the patient in bed for a correspondingly longer time.

